# Continuous Arabic Sign Language Recognition Models

**DOI:** 10.3390/s25092916

**Published:** 2025-05-05

**Authors:** Nahlah Algethami, Raghad Farhud, Manal Alghamdi, Huda Almutairi, Maha Sorani, Noura Aleisa

**Affiliations:** 1 Computer Science Department, College of Computing and Informatics, Saudi Electronic University, Riyadh 11673, Saudi Arabia; rghfarhud@gmail.com (R.F.); scigal80@gmail.com (M.A.); Hudaawadh13@gmail.com (H.A.); mdf.0213233132@gmail.com (M.S.); 2 Information Technology Department, College of Computing and Informatics, Saudi Electronic University, Riyadh 11673, Saudi Arabia; n.aleisa@seu.edu.sa

**Keywords:** Arabic Sign Language, TCN, BiLSTM, MediaPipe, sign language recognition

## Abstract

A significant communication gap persists between the deaf and hearing communities, often leaving deaf individuals isolated and marginalised. This challenge is especially pronounced for Arabic-speaking individuals, given the lack of publicly available Arabic Sign Language datasets and dedicated recognition systems. This study is the first to use the Temporal Convolutional Network (TCN) model for Arabic Sign Language (ArSL) recognition. We created a custom dataset of the 30 most common sentences in ArSL. We improved recognition performance by enhancing a Recurrent Neural Network (RNN) incorporating a Bidirectional Long Short-Term Memory (BiLSTM) model. Our approach achieved outstanding accuracy results compared to baseline RNN-BiLSTM models. This study contributes to developing recognition systems that could bridge communication barriers for the hearing-impaired community. Through a comparative analysis, we assessed the performance of the TCN and the enhanced RNN architecture in capturing the temporal dependencies and semantic nuances unique to Arabic Sign Language. The models are trained and evaluated using the created dataset of Arabic sign gestures based on recognition accuracy, processing speed, and robustness to variations in signing styles. This research provides insights into the strengths and limitations of TCNs and the enhanced RNN-BiLSTM by investigating their applicability in sign language recognition scenarios. The results indicate that the TCN model achieved an accuracy of 99.5%, while the original RNN-BiLSTM model initially achieved a 96% accuracy but improved to 99% after enhancement. While the accuracy gap between the two models was small, the TCN model demonstrated significant advantages in terms of computational efficiency, requiring fewer resources and achieving faster inference times. These factors make TCNs more practical for real-time sign language recognition applications.

## 1. Introduction

Sign language is the primary mode of communication for the deaf community; it relies on gestures, facial expressions, and body movements to convey meaning. Despite its complexity and richness, non-signers face significant challenges in understanding sign language, contributing to a persistent communication gap. Globally, over 1.3 billion people have hearing disabilities, according to the World Health Organization (WHO) [1]. Arabic Sign Language (ArSL) is under-resourced due to insufficient datasets and dedicated recognition systems. According to the 2022 Population and Housing Census, the Kingdom of Saudi Arabia has a total population of 32,175,224 people, with 1,349,585 individuals identified as having disabilities, representing 5.1% of the population. Among these, 84,025 individuals have hearing disabilities, accounting for approximately 6.2% of the disabled population [2]. In the broader Arab region, the prevalence of hearing disabilities varies significantly. A systematic review indicated that the incidence of hereditary hearing loss ranges from 1.20 to 18 per 1000 births annually. Notably, the prevalence was highest in Iraq at 76.3% and lowest in Jordan at 1.5% [3]. These statistics underscore the pressing need for accessible communication tools, such as Arabic Sign Language recognition systems, to bridge the communication gap for individuals with hearing impairments in Saudi Arabia and the wider Arab world.

Recognising Arabic Sign Language (ArSL) is critical to bridging communication gaps for the hearing and speech-impaired community. However, this domain has encountered significant hurdles that hinder its development and real-world application. One of the foremost challenges is the scarcity of comprehensive, high-quality datasets specific to ArSL. Unlike datasets for American or British Sign Language, which are often well-documented and openly available, resources for ArSL remain fragmented and limited. This lack of data restricts the ability to train robust models and hampers generalisation across diverse signers. Furthermore, the intricacies of Arabic Sign Language—such as unique handshapes, complex gestures, and the involvement of facial expressions—add another layer of difficulty, demanding innovative approaches in data collection and annotation [4].

Another critical challenge lies in the system’s ability to handle long-term temporal dependencies and signature style variability effectively. ArSL is dynamic, with gestures varying significantly between individuals due to regional dialects, personal habits, and expressive differences. Existing approaches, such as using Convolutional Neural Networks (CNNs) and Recurrent Neural Networks (RNNs), often fall short of capturing these nuances. For instance, systems trained on datasets like American Sign Language Recognition (ASLR) datasets have demonstrated accuracy drops when faced with signer variability or unseen gestures [5]. Addressing these challenges requires advanced temporal modelling techniques—such as Transformer-based models or Temporal Convolutional Networks (TCNs)—and an emphasis on real-world testing and adaptability [6].

With the advancement of machine learning, particularly in sequence modelling and video recognition, we aim to harness new technologies to improve communication accessibility for the Arab deaf community. Recent advancements in deep learning, such as Temporal Convolutional Networks (TCNs) [6,7] and Enhanced Recurrent Neural Networks (RNNs) with Bidirectional Long Short-Term Memory (BiLSTM) and attention mechanisms [4], have demonstrated potential in addressing these gaps, specifically for continuous sign language recognition.

The primary goal of our project is to recognise continuous Arabic Sign Language gestures from video sequences and interpret a sequence of signs rather than isolated gestures to enable more natural communication. To achieve this, we focus on comparing and optimising different model architectures—namely, Bidirectional Long Short-Term Memory (LSTM) with an attention mechanism and Temporal Convolutional Networks (TCN)—to determine which approach yields the highest accuracy and robustness in recognising continuous sign language sentences. Each model offers unique strengths: LSTMs are adept at handling sequential data through their memory mechanisms, while TCNs excel at capturing long-term dependencies through temporal convolutions.

This study is the first to use TCN models for Arabic Sign Language recognition. Through this pioneering approach, we hope to offer new insights into effective model architectures for sign language recognition and address the specific challenges associated with continuous Arabic Sign Language.

In the current study, the authors focused on recognising continuous Arabic Sign Language sentences from video sequences. The contributions can be summarised as follows:

- Dataset Creation: Developed a custom ArSL dataset and preprocess gesture sequences using MediaPipe key points.
- Model Development: Implemented TCN and enhanced RNN architectures for ArSL recognition.
- Performance Evaluation: Compared the models using accuracy, precision, recall, and robustness metrics.
- Optimisation: Fine-tuned the model hyperparameters to enhance performance in real-world scenarios.

## 2. Related Work

### 2.1. Machine Learning Models for Sign Language Recognition

Scholars have made significant progress in creating sign language interpreters in recent years. These systems correctly recognise and translate sign language motions into text using machine learning and deep learning algorithms [8,9]. A comprehensive review and analysis of machine learning methods for sign language recognition was conducted by Adeyanju et al. [10], highlighting the need for intelligent systems to improve recognition accuracy. It covers vision-based sign language recognition techniques, including image acquisition, preprocessing, segmentation, feature extraction, and classification. It emphasises the importance of ongoing research to develop more effective and inclusive sign language recognition systems. Kallingale and Prabu [11] designed a machine learning-based Sign Language Recognition (SLR) model for recognising isolated hand gestures, utilising a convex hull method for feature extraction and K-Nearest Neighbors (KNN) for classification. The model achieved an accuracy rate of 65%, with the authors emphasising that potential accuracy improvements can be achieved through larger datasets and different classifiers. Malik and Walia developed an advanced ML system for hand sign detection [12] to aid deaf people by applying computer vision and APIs. The system utilised transfer learning techniques, the SSD MobileNet V2 FPNLite 320 × 320 model, the TensorFlow Object Detection API, and the IBM cloud bucket. They attempted to train and deploy a model capable of recognising hand signs from video inputs, subsequently converting these into text and speech. The system achieved a precision rate of 69% and a recall rate of 70% on the test set, highlighting the model’s functionality through the web application.

Marzouk et al. [13] proposed a sign language recognition system using an artificial rabbits optimiser (ARO) with a Siamese neural network (SNN) for deaf people. They used the MobileNet model to extract feature vectors from sign language images, the SNN to classify the sign languages, and the ARO to adjust the parameters of the SNN. The dataset contained 1000 images of 10 classes of sign languages. The system achieved an average accuracy of 99.14%, a precision score of 95.72%, a recall rate of 95.70%, an F-score of 95.70%, and a G-measure of 95.71% on 500 epochs, outperforming several existing methods.

Another model was proposed by Sreemathy et al. [14] to distinguish continuous gestures via YOLOv4 (You Only Look Once) and an SVM (Support Vector Machine) model with MediaPipe. This model obtained an accuracy rate of 98.62% using MediaPipe and the SVM while obtaining a 98.8% accuracy rate using YOLO.

### 2.2. Deep Learning for Sign Language Recognition

Voulodimos et al. [15] provided a broad overview of deep learning, a branch of machine learning that employs artificial neural networks capable of learning from vast amounts of data. When used for sign language recognition and translation, deep learning models trained on sign language datasets show promise in converting signs into text or speech. The development of such models is pivotal for enhancing communication opportunities for the deaf and hard-of-hearing community, enhancing the expansive practical utility of deep learning technologies. In the scientific endeavour to improve communication opportunities for the deaf and hard-of-hearing members of the community, deep learning has emerged as a pivotal tool. Camgoz et al. [16] merged convolutional neural networks (CNNs), recurrent neural networks (RNNs), and attention mechanisms and introduced the RWTH-PHOENIX-Weather 2014T dataset to improve this domain with crucial resources. Their research demonstrated the potential of deep learning to improve translation accuracy using glosses for intermediaries and attention mechanisms. However, they also pointed out the inherent challenges of direct sign-to-text translation. Building upon Camgoz et al. [16], Al-Qurishi et al. [17] achieved accuracy rates above 95% for sign language recognition, although creating a universally reliable model remains challenging. In subsequent research, Murali et al. [5] pushed further boundaries by using a CNN-based system to recognise American Sign Language alphabets. Utilising web cameras and the HSV colour algorithm for hand gesture segmentation, they achieved an accuracy rate of over 90%, which underscored the transformative potential of CNNs in creating accessible communication tools for the deaf community by seamlessly harnessing deep learning advancements for practical applications.

Rastgoo et al. [18] expanded the literature on Sign Language Production (SLP) by examining the capabilities of deep learning to address the visual variability of signs and the intricate task of translating spoken and sign languages. Furthermore, Arekatla et al. [19] investigated deep learning and transfer learning for gesture detection to make sign language more comprehensible to non-signers. They proposed integrating voice assistance and text translation into multiple languages by broadening the accessibility of sign language communication. By deploying CNNs, ResNet50, and TensorFlow Object Detection, they showed how these techniques can be applied for sign language recognition, showcasing the potential of the technology to foster inclusivity.

Similarly, Nihalaani et al. [20] developed a sign language interpreter model that employs deep learning to translate sign language into text and speech, achieving an accuracy rate of 90.9%. The model validated the capacity of deep learning in aiding real-time sign language recognition and underscored its role in promoting inclusive communication. Extending these achievements, Triwijoyo et al. [21] presented a deep learning-based model for hand sign language recognition with an astonishing 99% accuracy rate. They used a seven-layer CNN trained on a vast dataset of 87,000 hand gesture images, setting a new standard for real-time hand sign recognition and offering a valuable resource for those learning sign language. A more advanced deep learning technique was introduced by Rayhan et al. [22], which demonstrated the pivotal role of Deep Residual Learning in revolutionising image and video recognition tasks. The research highlighted Residual Networks (ResNets) as a potential solution to deep neural networks’ vanishing/exploding gradient problem and degradation issues. By incorporating skip connections and residual blocks, ResNets enabled the training of ultra-deep networks, demonstrating superior performance across various benchmark datasets and highlighting the profound implications for Sign Language Translation (SLT) to potentially improve recognition accuracy and perform complex SLT tasks more effectively.

Lin et al. [6] proposed an enhanced model for recognising isolated word sign language using a combination of SKResNet and a Temporal Convolutional Network (TCN). The dataset used was LSA64, which contains 3200 videos of Argentine Sign Language. The SKResNet component reduces computation by dynamically selecting feature information from different perceptual fields, while the TCN captures temporal relationships between frames. The results showed a 100% accuracy rate on the LSA64 dataset, outperforming the traditional 3D-CNN and LSTM models. The key features include adaptive maximum pooling and the Mish activation function, improving generalisation and recognition efficiency. However, the limitations include challenges with continuous sign language recognition due to inconsistencies in natural speech.

Al Ahmadi et al. [6,7] introduced a pioneering hybrid model using a CNN-TCN tailored for automated sign language recognition. Their model amalgamated a Custom Convolutional Neural Network (CCNN) for intricate feature extraction alongside a Temporal Convolutional Neural Network (TCNN) for nuanced sequence modelling. They assessed this model utilising three standardised benchmark datasets encompassing both British and American sign languages, showcasing commendable performance metrics: a striking 95.31% accuracy rate, 94.03% precision rate, 93.33% recall, and a praiseworthy 93.56% F1-score across the datasets. The utilisation of the Custom Convolutional Neural Network (CCNN) and Temporal Convolutional Neural Network (TCNN) models, in conjunction with benchmark datasets featuring isolated letters and digits from British and American sign languages, underscored the efficacy of the proposed CNN-TCN model for sign language recognition, thereby emphasising its substantial potential within this specialised domain.

In summary, these studies underscored the transformative impact of deep learning on sign language recognition and translation, encouraging the development of more nuanced and accessible communication tools for the deaf and hard of hearing. By addressing the challenges of dataset diversity, model generalisation, and the complexity of translation tasks, these studies pave the way for future advancements that promise to make communication more accessible and inclusive for the deaf and hard-of-hearing communities.

### 2.3. Deep Learning Approaches for Arabic Dataset Recognition

Deep learning technologies have revolutionised sign language interpretation, enabling the development of more accurate and efficient communication solutions for the deaf community. This section provides a comprehensive overview of the recent developments in deep learning methods designed for Arabic Sign Language recognition. By addressing the unique challenges posed by Arabic Sign Language, such as the complex gestures commonly used in sentence structures and limited datasets, researchers have developed innovative models to enhance recognition accuracy and semantic translation capabilities. Their models helped identify individual signs accurately and interpret the overall meaning of a sequence of gestures, much like understanding the context and intent behind a sentence in spoken or written language.

Balaha et al. [4] introduced a Sign Language Recognition (SLR) system for Arabic Sign Language by utilising a dataset of 20 Arabic words; their deep learning architecture combines a Convolutional Neural Network (CNN) and Recurrent Neural Network (RNN) with bi-directional Long Short-Term Memory (BiLSTM) layers. The model achieved 98% accuracy on the dataset and notable results on the UCF-101 dataset, highlighting the proficiency of the model in recognising dynamic sign sequences. The study’s limitations included the need for validation on larger datasets and robustness testing against noise and environmental variations.

A novel semantic translation system for dynamic hand gestures was presented by Elsayed et al. [23], who integrated deep learning and ontology, particularly Multi-Sign Language Ontology (MSLO), which is a structured framework that organises and standardises the meanings, relationships, and concepts across multiple sign languages. MSLO helps unify the representation of signs to improve understanding, translation, and recognition across different sign languages. By leveraging Three-dimensional Convolutional Neural Networks (3D CNNs) and Convolutional Long Short-Term Memory (ConvLSTM), Elsayed et al. [23] created a gesture dataset consisting of 11 dynamic Arabic word gestures of one signer (dynamic refers to hand and body movements in Arabic Sign Language that involve continuous motion over time), and each sign was repeated 25 times. The model achieved a remarkable average accuracy rate of 97.4% across the three dynamic gesture datasets. GPU acceleration via Google Colab notably reduced the training time by approximately 87.9%. Integrating the translation system with MSLO facilitated semantic understanding and contextually relevant interpretations, enhancing recognition. Despite its success, challenges remained in handling complex gestures and ensuring user accessibility. This research marked a significant advancement in dynamic sign language recognition technology.

Bani Baker et al. [24] presented an Arabic Sign Language recognition (ArSL) system using convolutional neural networks (CNNs) to aid individuals with hearing impairments. The Sign Language dataset with 54,049 ArSL images for 32 Arabic alphabets was used. The researchers used six pre-trained combinations of models (vgg16, inceptionv3, resnet50v2, resnet152, Xception, and Mobilenetv2), which achieved a 98% accuracy rate without overfitting.

Another CNN model was developed by Abdelmoty et al. [25] using the ArSL dataset for the Arabic letters in sign language, achieving a 97.1% accuracy rate. The model utilises MediaPipe, a tracking framework for high-fidelity (highly detailed and precise representations) hand and finger gestures. MediaPipe prepares the dataset by extracting key points and inputting the sign images, feeding them to the CNN model with three hidden conventional layers, and then obtaining the text corresponding to the sign.

Ahmed et al. [26] developed a deep learning architecture using the ALGSL89 dataset containing 89 Algerian words. The model processed and examined the frames converted from a continuous video input into a series of frames in RGB. They used the MediaPipe library to extract the hand key points. The system was implemented using Python language and used deep learning libraries such as Tensorflow and OpenCv. The architecture for sign language recognition combines CNNs, LSTMs, and an attention mechanism within an Autoencoder framework. It consists of an encoder with two branches for processing video frames and hand landmarks, a bottleneck with an attention mechanism to focus on essential features, and a decoder employing LSTM networks to decode encoded features and classify sign language gestures. The Autoencoder achieved an accuracy rate of 98.99%.

Alsulaiman et al. [27] introduced a novel Convolutional Graph Neural Network (CGCN) architecture for sign language recognition tailored for the KSU-SSL database. The dataset consisted of numbers, alphabets, and words. This architecture incorporated separable 3DGCN layers and a spatial attention mechanism, addressing the common issue of over-smoothing in deep graph neural networks. The study highlighted the importance of creating a comprehensive dataset to facilitate the development of effective sign language recognition systems, aiming to improve communication for Saudi people with hearing disabilities. The study’s limitations include a restricted selection of 293 signs from 3000 available signs, variability in the data quality due to recording challenges, limited signer diversity, and the need for high computational resources, affecting the model’s generalizability and accessibility, as well as a dataset that is not publicly available.

Podder et al. [28] proposed an Arabic Sign Language (ArSL) recognition system designed for signer-independent scenarios, focusing on segmenting face and hand regions using MediaPipe Holistic to improve the model’s accuracy. The dataset includes realistic variations in signers’ gestures and appearances, and the video frames were preprocessed for consistency. The study evaluated multiple CNN-LSTM-SelfMLP architectures and showed that models trained on segmented datasets outperformed those using raw videos. Among the tested configurations, the MobileNetV2-LSTM-SelfMLP (q = 3) achieved the highest accuracy rate of 87.69%. The q value defines the complexity of the self-MLP classifier. At q = 1, the model has 4.98 million parameters, while at q = 3 and q = 5, 23,000 and 46,000 parameters were added, respectively. Higher q values enhance feature extraction without changing the number of layers or neurons. The authors noted the effectiveness of ROI segmentation and self-MLP layers in improving performance. They suggested expanding the dataset and incorporating attention mechanisms for future improvements in real-world applications.

In summary, a thorough review of the existing research reveals that while Temporal Convolutional Networks (TCNs) have not been applied to Arabic Sign Language recognition, its success in other sign languages suggests its potential for practical application. TCNs are highly effective for time-series analysis, as they efficiently capture long-range dependencies and process sequential data. These strengths make them particularly promising for recognising static or moderately complex gestures. However, scaling TCNs to larger datasets presents challenges such as overfitting, which can be alleviated through sparse attention mechanisms. Despite these limitations, the unexplored application of TCNs to Arabic Sign Language offers an exciting opportunity to contribute to the existing literature.

In addition, Enhanced Recurrent Neural Network (RNN) models featuring bi-directional LSTM and attention mechanisms are exceptionally well-suited for dynamic and continuous gesture recognition. These models leverage their bidirectional processing capabilities to analyse past and future context, which is crucial for tasks involving complex, sentence-level gestures. However, they are computationally intensive and require substantial resources to train effectively. Careful tuning is also necessary to prevent overfitting, especially given the scarcity of diverse Arabic Sign Language datasets.

Finally, one of the key challenges in Arabic Sign Language recognition is the limited availability of datasets, particularly for sentences and continuous gestures. The existing datasets often focus on isolated words or simple phrases, which fail to capture Arabic Sign Language sentences’ rich syntax and complexity. This limitation hampers the development of models capable of generalising from diverse and nuanced real-world scenarios. Addressing this gap by creating comprehensive, annotated datasets for sentence-level gestures is essential to advancing recognition systems in this field.

## 3. Methodology

**Figure 1:**
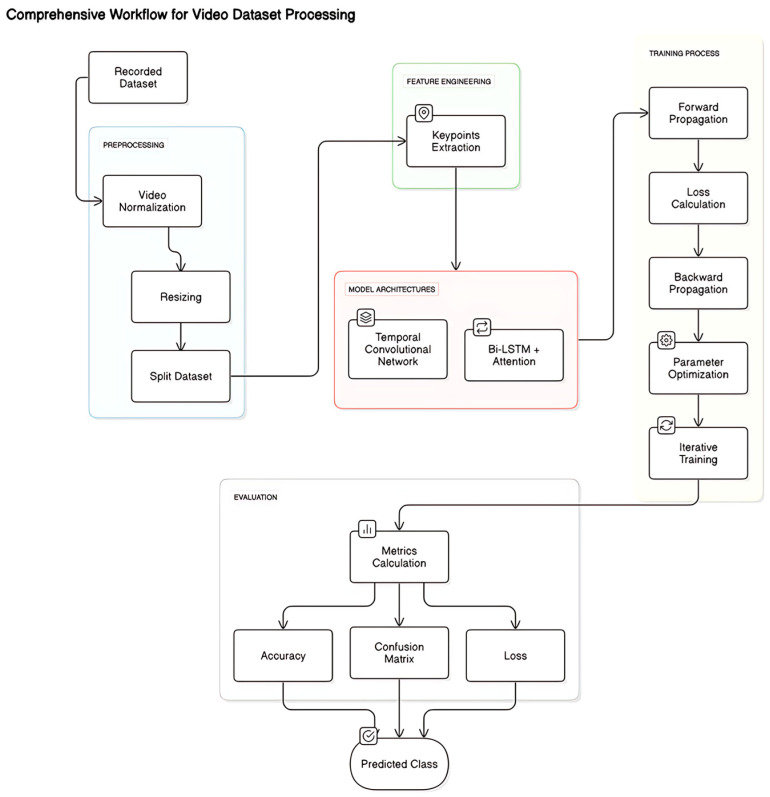
Methodology overview.

The methodology employed in our Arabic Sign Language Sentences (ArSLS) model is shown in Figure 1. It details the data collection, preprocessing, model architecture, and evaluation processes used for training and testing our deep learning models.

### 3.1. Data Collection and Preprocessing

To address the limitations of Arabic Sign Language datasets for sentences, we created a custom dataset comprising 3046 videos representing 30 distinct Arabic Sign Language Sentences (ArSLSs). These sentences are frequently used in Arabic Sign Language communication. Each sentence was repeated at least 100 times. Table 1 presents the sentences used in our dataset.

The selection of these 30 sentences was guided by real-world usage. As some members of our team have close relatives who are deaf or hard of hearing, we drew on firsthand knowledge of the expressions and phrases most commonly used in daily conversations. We also ensured the signs aligned with the formal Arabic Sign Language standards. This approach helped us choose practical sentences that are representative of natural communication in the deaf community.

Three signers performed the sentences in the dataset, ensuring consistency in the recording conditions. Each video lasted 4 s and was recorded at 30 frames per second (FPS) with a resolution of 640 × 480 pixels using a standard webcam. This ensured consistency across all samples. The dataset was structured to ensure consistency in labelling and format. Each video contained a person performing a sign corresponding to a specific sentence, and the sentences had similar grammatical patterns. The videos were stored in .mp4 format.

During the collection process, we maintained consistent lighting and background settings, and the videos featured multiple subjects performing the same sentences. This rich dataset was designed to address the scarcity of sentence-level Arabic Sign Language datasets, providing a robust foundation for training and evaluating the models.

The dataset was organised into folders, each representing a specific sentence. The structure of the dataset was as follows:

- Data_MP (Root folder containing key points data)
  - sentence_name_1 (folder for sentence 1)
    ▪ 0 (Video 1 folder)
    ▪ 1 (Video 2 folder)
    ▪ Additional videos as needed.
  - sentence_name_2 (folder for sentence 2)
    ▪ 0 (Video 1 folder)
    ▪ 1 (Video 2 folder)
    ▪ Additional videos as needed

### 3.2. Feature Engineering

We utilised MediaPipe to extract key points from hand and body landmarks for effective sign language recognition. These key points are crucial for the model’s performance and include the following features.

- Spatial Features: These capture static hand shapes and body postures, which were analysed in 1D space to understand the hand configurations at specific time steps.
- Temporal Features: These capture the movement of key points across frames, helping the model understand the dynamic nature of gestures over time.

The combination of spatial and temporal features enhances the ability of the model to interpret complex gestures and improve its overall performance and real-world applicability. The MediaPipe Holistic model was used [29]. MediaPipe detects both human pose and hand landmarks, which refer to key points on the fingers and palm that represent the position and movement of the hand. Each video frame was processed using MediaPipe to extract 33 pose key points and 42 hand key points (21 for each hand). Each key point provides spatial information, including (x, y, z) coordinates, a visibility score for pose key points, and (x, y, z) coordinates for hand key points. This results in 258 features per frame, which were calculated as shown below.

- Pose key points: 33 points × 4 values (x, y, z, visibility) = 132 features;
- Left hand key points: 21 points × 3 values (x, y, z) = 63 features;
- Right hand key points: 21 points × 3 values (x, y, z) = 63 features.

**Figure 2:**
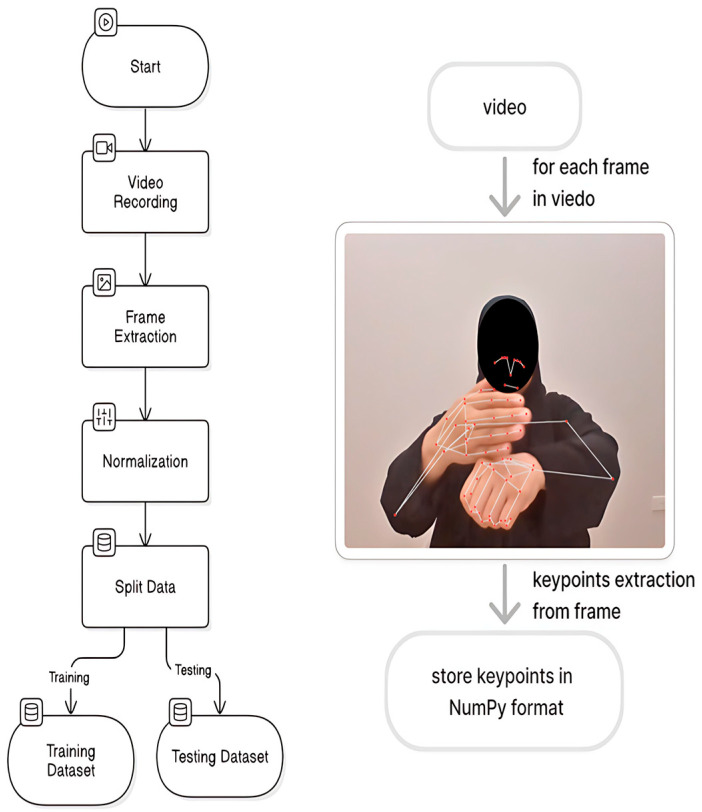
Key point extraction process.

These extracted features formed the input dimension for our models, and Figure 2 illustrates the key point extraction process.

Using key points rather than raw video frames has multiple advantages.

- Diversity and Robustness: This approach minimises the effect of variations in signers’ appearance, such as gender, clothing, skin tone, and lighting conditions. Focusing on skeletal and hand joint positions rather than visual features makes the model more robust to external and demographic variations.
- Lightweight and Efficient: Key point-based representation drastically reduces the input size and computational cost, enabling faster model training and inference. This is especially beneficial for real-time applications or deployment on devices with limited resources.

We built a more scalable and inclusive sign language recognition system by leveraging this lightweight and generalisable feature extraction technique.

### 3.3. Model Architecture

Two models were explored for recognising Arabic Sign Language: the Temporal Convolutional Network (TCN) and an Enhanced Recurrent Neural Network (RNN) with bi-directional LSTM and an attention mechanism. These models were chosen because they can effectively handle sequential data and capture temporal dependencies in sign language movements. TCNs are known for their ability to model long-range dependencies efficiently, while BiLSTM, with attention, enhances the contextual understanding of sequential data.

Other approaches, such as Transformer-based models and CNN-LSTM hybrids, could also be explored. However, we selected TCNs and BiLSTM due to their strong sequence modelling performance and suitability for structured sign language datasets.

#### 3.3.1. Temporal Convolutional Network (TCN)

Temporal Convolutional Networks (TCNs) are an architecture designed specifically for sequential data processing. They replace recurrent layers in traditional architectures like RNNs and LSTMs with convolutional layers, making them highly suitable for sign language recognition [6]. In the context of our Arabic Sign Language Recognition (ArSLSR) project, the TCN model can capture both short-term and long-term dependencies in the key point sequences extracted from the videos.

**Figure 3:**
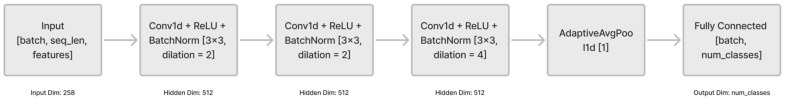
TCN architecture.

Temporal Convolutional Networks (TCNs) employ 1D dilated convolution layers, which expand the receptive field by skipping a fixed number of input points, allowing the model to capture long-range dependencies in sequence data more efficiently [6,7]. This approach enables TCNs to process temporal information without relying on recurrent connections. Using causal convolutions, TCNs maintain the temporal order of inputs, ensuring that only past and present time steps influence predictions. A significant advantage of TCNs is their use of dilated convolutions, which allow the model to cover more extended sequences without requiring deeper networks, thus enhancing computational efficiency. Additionally, residual connections can be incorporated to mitigate the vanishing gradient problem, ensuring stability during training. The final output layer typically consists of a Softmax function or a fully connected layer. The Softmax function transforms a vector of raw scores (logits) into a probability distribution, where each value represents the likelihood of the input belonging to a specific class. It ensures that all probabilities sum to 1, making it particularly useful for multi-class classification tasks, which map the learned features to the desired predictions. Figure 3 shows the TCN architecture. This architecture is computationally efficient and well-suited for recognising long-duration sign language gestures, as was demonstrated in recent studies (e.g., Lin et al., [6]; Al Ahmadi et al. [7]). TCNs utilise dilated convolutions, which allow them to model long-range dependencies in sequence data with fewer parameters and reduced computational complexity compared to traditional RNN-based models. These features make TCNs particularly effective for recognising long-duration gestures without compromising performance.

##### Key Architectural Components

1. 1D Convolutions:

- TCNs use 1D convolutional layers rather than recurrent layers. This allows the model to process sequential data more efficiently by capturing patterns over time [7].
- Unlike recurrent architectures, which process data step by step (one time step at a time), TCNs apply convolutions to the entire sequence simultaneously by using dilated convolutions. This allows the model to consider the whole input sequence in parallel instead of sequentially processing each time step, significantly speeding up the computation and making it easier to scale.

2. Causal Convolutions:

- Causal convolutions ensure that each time step’s prediction only depends on the previous time steps, preserving the temporal order crucial for sign language recognition.
- This is especially important for gesture sequences, where the order of actions is crucial.

3. Dilations:

- Dilated convolutions were used to expand the receptive field of the model. By applying convolutions at intervals, TCNs capture long-term dependencies efficiently without needing recurrent connections.
- This enables the model to look across the entire sequence, even in long sequences, making it ideal for recognising sign language, where some gestures involve long temporal dependencies.

4. Residual Connections:

- Residual connections help train deeper networks by stabilising the gradient flow. They prevent issues like vanishing gradients that could occur during training and enhance the model’s capacity to learn more complex representations.
- These connections are essential in preventing performance degradation in deeper models.

##### Working Principle

In this architecture, input sequences of the key points representing body joints and gestures pass through several convolutional layers. Each layer uses increasing dilation factors, allowing the model to capture patterns across various time scales. By leveraging these convolutional layers, the TCN can recognise subtle differences in Arabic Sign Language gestures and posture variations.

For instance, the model learns to distinguish between similar sentences by capturing the unique temporal dynamics of each sign. It maps these gestures to specific Arabic sentences using hierarchical feature extraction across convolutional layers.

##### Model Structure

1. Input and Output Dimensions:

- The model is initialised with input_dim (number of features per time step), hidden_dim (number of channels in the convolutional layers), and num_classes (number of output classes for classification).

2. Convolutional Layers:

- The TCN uses three one-dimensional convolutional layers (Conv1d), each configured with a kernel size of three and increasing dilation rates (2, 2, and 4).
- Dilation allows the model to effectively capture long-range dependencies by increasing the receptive field without needing deep networks.
- The padding ensures the output sequence length remains consistent with the input sequence.

3. Activation and Normalisation:

- Each convolutional layer is followed by a ReLU activation function to introduce non-linearity.
- Batch normalisation (BatchNorm1d) is applied after each activation to stabilise training and improve performance.

4. Global Average Pooling:

- After the convolutions, an adaptive average pooling layer (AdaptiveAvgPool1d) is used to compress the temporal dimension into a single value per feature channel, reducing the sequence to a fixed size.

5. Fully Connected Layer:

- The output of the pooling layer is passed through a fully connected (Linear) layer to map the features to the number of classes for classification.

##### Data Flow

1. Input Permutation:

- Since the TCN expects the input in the format [batch_size, channels, sequence_length], the input tensor is permuted from [batch_size, sequence_length, features] to match this requirement.

2. Convolution and Pooling:

- The permuted input is passed through the sequence of convolutional layers, with each layer learning to extract temporal patterns.

3. Dimensionality Reduction:

- After pooling, the output tensor is squeezed to remove the redundant temporal dimension, leaving a tensor with shape [batch_size, hidden_dim].

4. Final Prediction:

- The tensor is passed through the fully connected layer to produce predictions for each class.

##### Advantages of TCN

- Efficiency: TCNs are computationally more efficient than RNNs or LSTMs, especially when handling long sequences. This efficiency is crucial when processing large datasets like our Arabic Sign Language video data [7].
- Long-range Dependencies: Dilated convolutions enable TCNs to capture long-range dependencies in sequence data without requiring recurrent connections. This helps maintain high performance while allowing for more efficient processing of long-duration inputs.
- Causal Nature: The causal convolutions ensure that the model respects the temporal order of gestures in sequence data. In other words, each output of a convolution layer only depends on the current and previous time steps, not on future ones. This is crucial for tasks like sign language recognition, where the sequence of gestures must be interpreted correctly to understand the meaning accurately.

#### 3.3.2. Enhanced RNN with Bi-Directional LSTM and Attention

**Figure 4:**
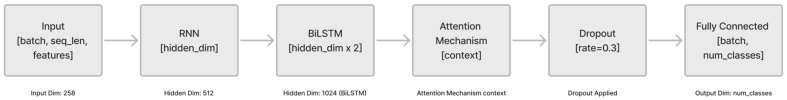
Enhanced RNN with BiLSTM and attention mechanism architecture.

The Enhanced Recurrent Neural Network (RNN) with bi-directional LSTM and an attention mechanism utilise multiple layers to capture complex dependencies within sequential data. By processing input sequences in both the forward and backward directions, the bi-directional LSTM improves context capture, enriching the model’s understanding of sign language gestures. The attention mechanism further enhances performance by allowing the model to focus on the most relevant segments of the input sequence, computing attention weights that prioritise key elements in continuous sign language gestures. This architecture typically includes stacked bi-directional LSTM layers [4] and dropout layers to prevent overfitting. It concludes with a fully connected layer that transforms processed features into the desired output, as shown in Figure 4. This model benefits from capturing long-term dependencies and focusing on the most informative parts of the sequence.

##### Architecture Overview

1. Bi-Directional LSTM:

- A regular LSTM layer processes the sequence in a single direction, from the first frame to the last. A BiLSTM layer extends this by processing the sequence in the forward and backwards directions. This means that it can consider the past frames and the future frames when predicting a gesture, capturing a broader context.
- This is particularly useful in sign language recognition, where the meaning of a gesture may depend on both the previous and upcoming movements.

2. Attention Mechanism:

- The attention mechanism allows the model to assign more importance (higher weights) to specific frames in a sequence that are more informative or discriminative for the task.
- For example, not all movements are equally important in a sign language sentence. Some frames contain critical gestures that distinguish one sign from another, and the attention mechanism helps the model focus on those keyframes.

##### Working Principle

- The input key point sequence is first passed through a BiLSTM layer, which processes the temporal data in the forward and backward directions. This gives the model a more comprehensive understanding of the sign language movements.
- After the BiLSTM layer, the attention layer assigns importance weights to different frames in the sequence. This is crucial for focusing on frames with more relevant gestures or transitions.
- Finally, the weighted frames are aggregated, and the model predicts the sentence based on this focused information.

##### Model Structure

The Enhanced RNN with bi-directional LSTM and attention model was structured using two technologies: an attention mechanism, whose output is fed into the enhanced BiLSTM model.

##### Attention Mechanism

- Purpose: The attention mechanism allows the model to focus on the most relevant parts of the input sequence by assigning weights to each time step.
- Implementation:

▪ A linear layer (nn.Linear) calculates attention scores for the hidden states from the BiLSTM output.
▪ These scores are transformed using the tanh activation function and normalised with Softmax to obtain the attention weights.
▪ The attention weights are applied to the LSTM output using batch matrix multiplication (torch.bmm), creating a context vector. This vector summarises the sequence by focusing on the most essential features.

##### Enhanced BiLSTM Model Structure

1. Input Dimensions

- input_dim: Number of features in each time step.
- hidden_dim: Number of units in the hidden layers.
- num_classes: Number of output classes.

2. RNN and BiLSTM Layers

- An RNN layer processes the input to capture initial temporal dependencies.
- A bi-directional LSTM layer follows, capturing both forward and backward temporal information, which enhances the model’s ability to understand sequential data.

3. Attention Layer

- The BiLSTM outputs are passed to the attention mechanism, which calculates a context vector summarising the sequence.

4. Dropout Layer

- Dropout regularisation is applied to the context vector to prevent overfitting.

5. Fully Connected Layer

- The context vector is passed through a fully connected (Linear) layer, which maps the features to the output classes.

##### Data Flow

1. RNN and BiLSTM Processing:

- The RNN first processes the input sequence to extract initial temporal patterns.
- The output of the RNN is passed to the BiLSTM layer to capture bidirectional information.

2. Attention Application:

- The attention mechanism assigns weights to each time step in the BiLSTM output, emphasising the most critical parts of the sequence.

3. Context Vector and Classification:

- The attention mechanism generates a context vector, a weighted summary of the BiLSTM outputs.
- After dropout, the context vector is input to the fully connected layer to produce the final class predictions.

### 3.4. Training Procedure

The training process involved several stages to optimise the models for sign language recognition.

- Data Preparation: The dataset was preprocessed by normalising, resizing, and splitting it into training and test sets.
- Model Building: The TCN and Enhanced RNN models were constructed with their respective layers, optimisers, and loss functions.
- Initialisation and Forward Propagation: The initial parameters were set, and input sequences were processed through the respective models.
- Loss Calculation and Backward Propagation: The loss using categorical cross-entropy, updated model weights, and backpropagation was computed.
- Parameter Update: The weights were updated using optimisers like Adam or SGD based on the computed gradients, with learning rates controlling the update sizes.
- Iterative training: The models underwent multiple epochs (an epoch is one complete pass through the training data during model training), refining their understanding through repeated forward and backward propagation, gradually improving their performance.
- Evaluation: The models were evaluated using accuracy, F1-score, and other relevant metrics to assess performance and determine the best model.

### 3.5. Implementation Details and System Setup

The experiments were conducted on a 64-bit operating system with an Intel Core i7-10510U CPU @ 1.80 GHz, 16 GB of RAM, and an NVIDIA GeForce MAX250 GPU. This setup provided sufficient resources for training deep learning models efficiently.

Both models—the TCN and the Enhanced BiLSTM with attention—were implemented using PyTorch (v2.0.1) and trained on the NVIDIA GPU. Development and visualization were carried out using Jupyter Notebook. The input feature vector consisted of 258 dimensions, extracted from sign language videos using MediaPipe (v0.9.1). The hidden dimension was set to 512, and the output layer predicted one of 30 sentence classes from the ArSLSR dataset.

For the TCN model, three 1D convolutional layers were employed with a kernel size of 3, padding of 2, and dilation rates of 2, 2, and 4, respectively. Each convolutional block included ReLU activation and batch normalization. The final classification was preceded by an adaptive average pooling layer. The model used the Adam optimizer with a learning rate of 0.001.

For the Enhanced BiLSTM model, the architecture began with an RNN layer followed by a bi-directional LSTM, utilizing 512 hidden units. An attention mechanism was applied after the LSTM output, followed by a dropout layer with a rate of 0.3. The model was trained using the AdamW optimizer with a learning rate of 0.001.

Both models were trained for 30 epochs with a batch size of 8 using Cross-Entropy Loss as the loss function.

Additional tools and libraries included:

- NumPy (v1.24.2) for numerical operations and preprocessing.
- Scikit-learn (v1.2.2) for data splitting and normalization.
- Matplotlib (v3.7.1) and Seaborn (v0.12.2) for visualization, including confusion matrices.

### 3.6. Evaluation Metrics

The following metrics were used to evaluate model performance.

- Accuracy: The proportion of correctly classified predictions.
- Precision: The proportion of true positive predictions among all positive predictions.
- Recall: The proportion of true positive predictions among all actual positive instances.
- F1-Score: The harmonic means of precision and recall, balancing both metrics.
- Confusion Matrix: A breakdown of the true positives, false positives, true negatives, and false negatives for each class, providing deeper insights into model performance.
- These metrics collectively allow for a comprehensive assessment of the ability of the model to recognise sign language gestures effectively [30].

## 4. Result and Analysis

The testing phase assessed the system’s functionality, effectiveness, and adaptability. Three key experiments were conducted to evaluate performance across the various scenarios. Evaluation metrics, including confusion matrices, accuracy scores, precision, recall, and F1-score, were used to analyse the results (as described in Section 3).

### 4.1. Evaluation Methods

#### 4.1.1. Evaluation of the Models Using the ArSLSR Dataset

The first experiment aimed to evaluate model performance on the primary dataset, ArSLSR, which was used for training and testing. The dataset consists of 3046 videos representing 30 Arabic Sign Language sentences. The data were split by 80% for training (2436 videos) and 20% for testing (609 videos).

**Figure 5:**
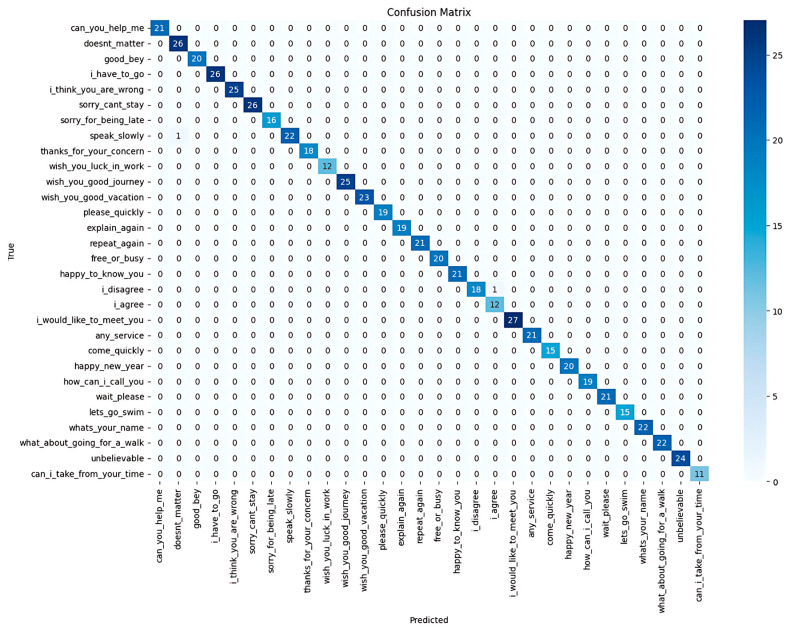
Confusion matrix for TCN model on our dataset (ArSLSR).

The model achieved an overall accuracy of 0.995, with a precision of 0.99, a recall of 1.00, and an F1-score of 0.99. The confusion matrix (Figure 5) showed minimal misclassifications, mainly between visually similar gestures.

#### 4.1.2. Evaluation of the Models Using Non-Signer Videos (Unseen Data)

Evaluations were conducted using videos recorded by three non-signers not involved in the original data collection to test the model’s real-world applicability and adaptability. These individuals included a middle-aged male, a middle-aged female, and a teenage girl. Notably, the first two participants are members of the deaf community.

Each participant was asked to perform five sentences from the 30 classes in our ArSLSR dataset, resulting in 15 evaluation videos. This evaluation aimed to examine whether the model could generalise effectively to unseen individuals while maintaining a high prediction accuracy for familiar sign classes.

To facilitate this testing process, we developed a lightweight evaluation tool that allows users to upload a video and receive a prediction from the trained TCN model. The tool outputs the predicted sign class and a confidence score (the highest probability value from the model’s Softmax layer), indicating the model’s certainty in its prediction. Higher confidence values (closer to 1) reflect greater certainty, while lower values indicate less confidence.

In our evaluation of the TCN model, all the predictions across the 15 videos were accurate and accompanied by high confidence scores (ranging from 0.85 to 1), suggesting a strong correlation between confidence and prediction correctness.

**Figure 6:**
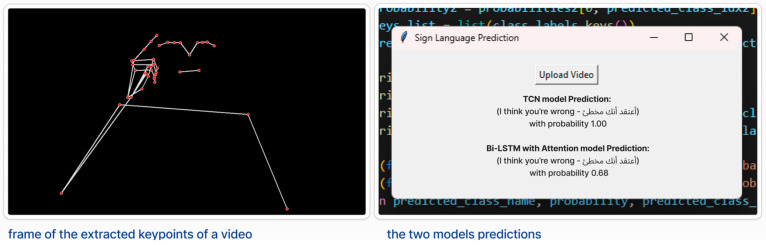
Screenshot of test example. The left image shows extracted keypoints from a video, where red dots represent the detected joints (e.g., hands, elbows), and white lines indicate the connections between them. The right image displays predictions from the two trained models. The figure shows the prediction for the sentence (I think you’re wrong) which predicted correctly in both models.

Figure 6 shows a screenshot of the testing interface. Table 2 presents a sample of five results from the 15 evaluated videos. All the predictions across the 15 videos were accurate and accompanied by high confidence scores, demonstrating the model’s strong generalisation performance across different signers.

#### 4.1.3. Evaluation of TCN Model Using External Dataset (ArabSign)

The third experiment evaluated the model’s generalisation ability using a different Arabic Sign Language dataset, ArabSign, which was created by Luqman and Elalfy [31]. This dataset comprises 9335 videos representing 50 ArSL sentences performed by six signers. Due to hardware limitations, only 27 sentences (4965 videos) were used in our experiments, with 80% used for training (3972 videos) and 20% for testing (993 videos).

**Figure 7:**
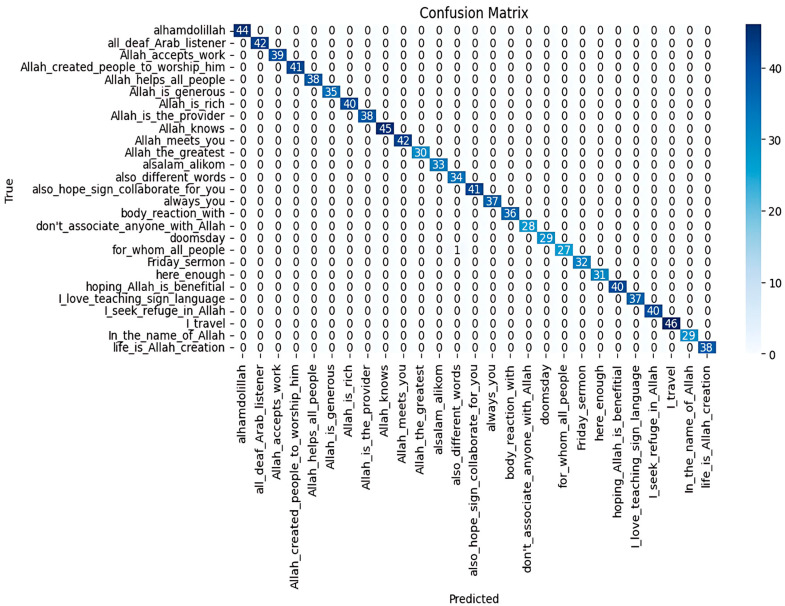
Confusion matrix for the evaluation of TCN model on an external dataset.

The model achieved an overall accuracy of 0.99 and precision, recall, and F1-scores of 0.99, demonstrating consistent performance even with an external dataset. The confusion matrix is shown in Figure 7.

### 4.2. TCN Model Evaluation

#### 4.2.1. Evaluation of TCN Model Using Our Dataset (ArSLSR)

The TCN model was trained and tested on the ArSLSR dataset containing 30 Arabic Sign Language sentences. The total number of dataset videos was 3046; 80% (2436 videos) was used for model training, and 20% (609 videos) was used as test samples. The model achieved an impressive overall accuracy of 0.995, indicating that nearly all the predictions were correct. The precision rate was 0.99, highlighting the ability of the model to minimise false positives across all classes. Additionally, the recall reached 1.00 since the model successfully identified all instances of each class without missing any. The F1-score of 0.99 indicates a strong balance between precision and recall, ensuring consistent performance across the dataset. With 609 samples evaluated, the model’s high performance shows its reliability and effectiveness in correctly classifying most signs, with only minimal errors observed.

The confusion matrix (Figure 5) further supports these conclusions, showing that most predictions aligned with the truth, with only a few misclassifications. Notably, these misclassifications occurred between sentences with visually similar gestures, indicating a potential area for improvement in handling subtle differences in signing. Overall, the performance of the TCN model demonstrated its robustness and reliability for sign language recognition.

#### 4.2.2. Evaluation of TCN Model Using New Test Videos (Unseen Data)

The TCN model was evaluated using videos from people not included in the dataset collection to test its real-world applicability. To facilitate the test process, we built a simple test code to allow us to upload a video and easily evaluate the system’s predictions. The process was as follows:

- Upload a video containing one of the classes included in the ArSLSR dataset.
- Use the TCN model to predict the sign class.
- Obtain output of predicted class and confidence score for the model.

Figure 6 shows a screenshot of the testing process.

As shown in Table 2, the TCN model accurately predicted all the input sentences with high confidence, demonstrating robust adaptability.

#### 4.2.3. Evaluation of TCN Model Using External Dataset

The TCN model was trained and tested on samples from a publicly available Arabic Sign Language dataset (ArabSign) [31] containing 27 out of the 50 sentences in the dataset.

The model achieved an impressive overall accuracy of 0.99, indicating that nearly all the predictions were correct. The model also scored 0.99 for precision, recall, and F1-score, ensuring consistent performance across the dataset confusion matrix, as shown in Figure 7.

### 4.3. Enhanced BiLSTM with Attention Model Evaluation

#### 4.3.1. Evaluation of BiLSTM with Attention Model Using Our Dataset (ArSLSR)

**Figure 8:**
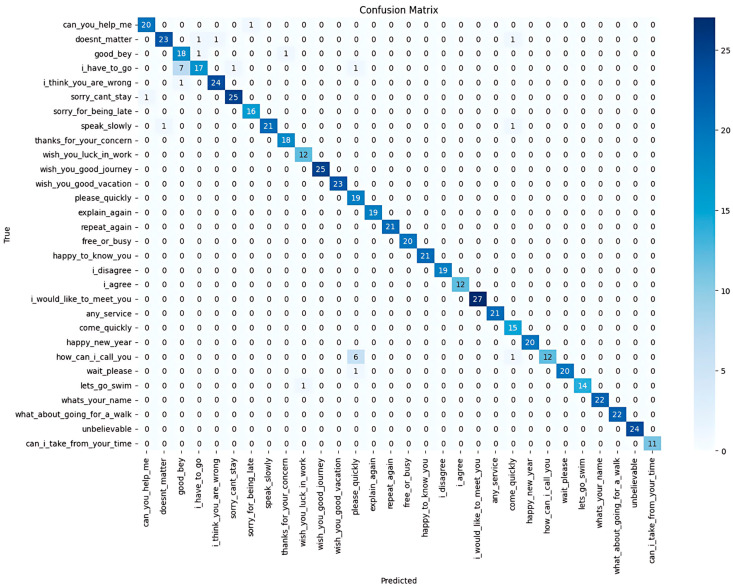
Confusion matrix for Enhanced BiLSTM with attention model’s performance on our dataset (ArSLSR).

The BiLSTM with attention model was trained and evaluated on the ArSLSR dataset, which includes 30 Arabic Sign Language sentences. The model achieved an accuracy rate 0.96, indicating a strong overall performance. The precision rate of 0.97 shows that the model effectively minimised false positives, while the recall rate of 0.96 highlights that it accurately identified most instances of each class. The F1-score of 0.96 reflects a good balance between precision and recall, ensuring reliable classification performance across the dataset. The confusion matrix further confirmed that the model correctly classified most signs, with some misclassifications occurring between visually similar gestures, which is an area for potential improvement (Figure 8).

#### 4.3.2. Evaluation of BiLSTM with Attention Model Using New Test Videos (Unseen Data)

We tested the BiLSTM with attention model on videos from unseen data to assess the model’s real-world applicability. This evaluation used the same simple test code used for the TCN model that allowed for easy uploading of videos and prediction of the sign classes.

As shown in Table 3, the model accurately predicted most input sentences with high confidence, with one misclassification with low confidence. The misclassification observed in the BiLSTM with attention model likely stemmed from insufficient generalisation to unseen data due to the limited diversity in the training set, especially for complex or underrepresented sentences like “أتمنى لك التوفيق في العمل”. Overlapping features between similar classes, variability in signing styles, and potential misalignment of the attention mechanism further contributed to errors, as the model may have focused on irrelevant parts of the gesture sequence. The low confidence score of 0.65 for the misclassified sentence reflects uncertainty, potentially due to poor calibration between the confidence and true accuracy. In contrast, correctly predicted sentences with high confidence suggest that the model performed well for well-represented and distinct classes, where the attention mechanism accurately captured the key features.

#### 4.3.3. Evaluation of BiLSTM with Attention Model Using External Dataset

The BiLSTM with attention model was trained and tested on 27 sentence samples out of the 50 in the ArabSign dataset; 80% was used for training (3972 videos), and 20% was used for testing (993 videos).

Initially, the BiLSTM model performed poorly on the external dataset, with an accuracy rate of just 0.26. However, several enhancements were made to the model architecture and training process that significantly improved its performance. These improvements led to the model achieving a much better accuracy rate of 0.97. The following is a breakdown of the enhancements that were implemented:

1. Addition of a 1D Convolutional Layer

- The Conv1d layer was used as a feature extractor to detect local patterns in the temporal input sequences.
- It reduces noise and enhances the representation of features passed to the RNN, resulting in more meaningful input for downstream layers.
- This helps the model learn spatial and temporal hierarchies early in the pipeline.

2. Increased the Depth in the BiLSTM Layers

- Deeper BiLSTMs can capture more complex dependencies in sequential data to allow the model to understand higher-order temporal patterns, improving its ability to represent long-term dependencies.

3. Explicit Weight Initialisation

- Proper weight initialisation ensures faster convergence during training and prevents problems like vanishing or exploding gradients, which likely improves stability and training efficiency.

4. Learning Rate Adjustment

- A lower learning rate provides more fine-grained weight updates, which is especially important for deeper architectures to avoid overshooting minima during optimisation.
- This change contributes to more stable and practical training.

5. Structural Adjustment in Input Handling

- This ensures compatibility with the convolutional layer and maintains temporal order.
- This adjustment allows the model to effectively use the added convolutional features.

**Figure 9:**
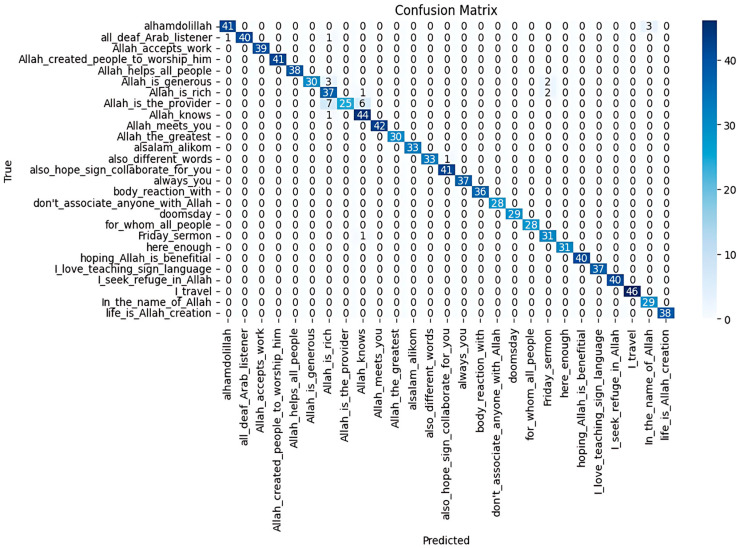
Confusion matrix for Enhanced BiLSTM with attention model performance on the external dataset.

The enhancements made to the BiLSTM model dramatically improved its performance. With an accuracy rate of 0.97, precision rate of 0.98, recall rate of 0.97, and an F1-score of 0.97, the model now exhibited a robust ability to recognise sign language gestures. The confusion matrix further confirmed that the model had a low rate of misclassification, ensuring high reliability in real-world applications (Figure 9).

#### 4.3.4. Evaluation of the Enhanced Model on Our Dataset (ArSLSR)

After applying the enhancements to the BiLSTM model, we retrained it on our dataset to evaluate how these changes impacted its performance in the context of the training data. The results were promising, with a noticeable increase in all key metrics. The model’s accuracy increased from 0.96 to 0.99, with the precision, recall, and F1-score values all reaching 0.99.

### 4.4. Evaluation of Unknown Gestures Outside the Predefined Classes

To ensure robustness and reliability in real-world applications, we conducted an additional experiment to test the model’s response to gestures outside the predefined 30 classes in the ArSLSR dataset. Specifically, we tested the model using 10 videos of gestures that are not part of the 30 predefined classes in the ArSLSR dataset. These videos were recorded separately for this experiment. We aimed to assess whether the model could identify and flag unknown or out-of-distribution (OOD) gestures rather than confidently misclassify them.

A condition was implemented in the prediction phase that checks the confidence score of the model’s output. If the confidence score falls below a defined threshold (e.g., 0.60), the system outputs the result as “Unknown Gesture” and a message indicating that it can only recognise predefined sign language sentences from the dataset.

#### 4.4.1. Testing Procedure

- Input videos with gestures not included in the original 30 classes were recorded.
- These videos were passed through the same preprocessing pipeline and fed into the trained models.
- If the prediction confidence score was low, the result was flagged as “Unknown Gesture”.

#### 4.4.2. Observations

- The model successfully produced low-confidence scores for most of the unknown input gestures.
- In such cases, the system did not force a class prediction but instead triggered the unknown gesture output, showing the message, “The system cannot recognise this gesture. Please use one of the predefined sign language sentences”.
- For example, a gesture not belonging to the 30 classes resulted in the following messages:
  ▪ Predicted class: There is no match.
  ▪ Confidence Score: 0.42.
  ▪ Output Message: “Unknown gesture. This system supports only predefined sign language classes”.

#### 4.4.3. Significance

This test demonstrated that the system can handle unexpected inputs by using confidence-based filtering. Such behaviour is crucial for real-world usability to avoid misleading outputs or incorrect translations when the system encounters unfamiliar signs.

#### 4.4.4. Future Improvements

- Training with additional “unknown” class examples to better model OOD gestures.
- Incorporating uncertainty estimation methods like Monte Carlo Dropout or entropy-based confidence scoring to further enhance unknown gesture detection.

### 4.5. Discussion

The evaluation of the Temporal Convolutional Network (TCN) and the Enhanced BiLSTM with attention model provided valuable insights into the effectiveness of these models for Arabic Sign Language recognition. By testing the models on multiple datasets—our own ArSLSR dataset, videos from non-signers, and an external dataset—we highlighted the strengths and weaknesses of each approach.

The TCN model was designed to capture local temporal dependencies in the sequence data through its convolutional layers. This design enables it to perform well on the dataset, showcasing strong results across in-domain (ArSLSR) and out-of-domain (external dataset) data. On the other hand, the BiLSTM model, before the enhancements, struggled with understanding temporal patterns, leading to poor performance on the external dataset. The upgrades, however, allowed the model to learn more effectively by incorporating local feature extraction (via Conv1D) and global context (via BiLSTM and an attention mechanism).

Another notable difference was observed in the evaluation of non-signer videos. Both models performed well, but the BiLSTM model showed slightly lower confidence in some instances, particularly for sentences with visually similar signs. This suggests that more work could be performed to enhance its discriminative power.

Table 4 summarises the TCN and Enhanced BiLSTM findings with attention models across the various evaluation metrics and scenarios, while Table 5 presents a direct comparison of key performance differences between the two models.

The testing and evaluating of the TCN and Enhanced BiLSTM with attention models demonstrated the effectiveness of these deep learning architectures for sign language recognition. The TCN model performed exceptionally well on the ArSLSR and external datasets, making it reliable for recognising sign language gestures. After significant modifications, the Enhanced BiLSTM with attention model also demonstrated strong performance, especially after retraining on the ArSLSR dataset, achieving near-perfect metrics.

The improvements made to the BiLSTM model were crucial for unlocking its potential, and the results suggest that combining both models could yield even better performance. However, challenges remain in distinguishing between visually similar gestures and improving real-time recognition capabilities. Future work could address these challenges by exploring more advanced techniques for data augmentation and integrating real-time translating systems for practical sign language translation applications.

Overall, both models proved to be highly capable of recognising Arabic Sign Language, with the Enhanced BiLSTM model showing significant promise for real-world applications. However, the simplicity and the high performance of the TCN model make it an excellent choice for sign language recognition tasks.

## 5. Conclusions

The Arabic Sign Language Recognition project is a significant step forward in fostering independence for the deaf and hard-of-hearing community. By enabling effective communication in critical environments such as restaurants, airports, and hospitals, our system aims to eliminate third-party intervention in facilitating conversations. The project leverages deep learning models like Temporal Convolutional Networks (TCNs) and Enhanced BiLSTM with an attention mechanism. It incorporates innovative methodologies, such as key point extraction, to overcome privacy concerns and data collection barriers. This approach ensures a scalable, privacy-conscious solution that can be extended to multiple applications.

By comparing the Temporal Convolutional Network (TCN) model and the Enhanced BiLSTM with the attention model, we demonstrated that the TCN model excelled in generalisation, speed, and accuracy across the internal and external datasets, achieving an accuracy of 0.995 on the ArSLSR dataset and 0.99 on the external data. Although the TCN model’s simplicity and robust temporal modelling resulted in consistent performance and high prediction confidence, it faced minor challenges with visually similar gestures. Conversely, the BiLSTM with attention model showed remarkable improvement following enhancements, increasing its external dataset accuracy from 0.26 to 0.97. Still, it remained slower and slightly less reliable than the TCN model in some cases.

The novelty of this project lies in its pioneering application of Temporal Convolutional Networks (TCNs) to Arabic Sign Language recognition, becoming the first study, to our knowledge, to explore TCNs in this context. Additionally, the project addresses privacy concerns by not storing raw videos in the dataset and relying on extracted key points, ensuring the original visual data remains secure. This privacy-oriented approach highlights the potential of leveraging key points to build robust models while safeguarding user data.

Furthermore, our dataset was created using simple hardware like webcams, demonstrating how low-resource environments can contribute to impactful AI development. The democratisation of data creation and model training paves the way for similar advancements in other domains, emphasising the potential for innovation in underrepresented languages and contexts.

## 6. Future Work

Although the current model has proven effective, several avenues exist for future enhancement. One of the primary challenges in sign language recognition remains the poor availability of comprehensive datasets that cover the breadth of signs and dialects used across different regions. Our project highlights the need for more collaborative efforts in building diverse and inclusive datasets. To address this, we propose developing a customisable application or web platform that allows users to create and contribute to public datasets.

Such a platform would empower the members of the deaf community by encouraging them to record their sign language gestures and add corresponding labels. The system could include integrated pipelines for key point extraction and model training, enabling users to build personalised models tailored to their dialects and preferences. This adaptability would demonstrate the richness of sign language dialects and reduce the need for standardisation, which can be restrictive for users.

Future work could incorporate data augmentation techniques to simulate realistic noise conditions and evaluate the model’s performance under such variations to enhance the model’s adaptability and generalisation in uncontrolled environments.

Additionally, leveraging generative AI models represents a significant opportunity for the future of Arabic Sign Language recognition. These models could be trained to generate synthetic Arabic Sign Language datasets, simulating gestures for words and sentences not present in the original dataset. Such an approach would help overcome dataset scarcity while ensuring diversity and inclusivity in training data.

Furthermore, future work can explore advanced model architectures, such as transformers and hybrid models combining temporal convolution with self-attention, to improve accuracy and generalisability. Exploring hardware-specific optimisations, such as deploying the model on lightweight devices or edge systems, could enhance accessibility and usability for real-world applications.

We also plan to incorporate cross-validation as part of future experiments to strengthen the statistical reliability of our results. This will allow for a more rigorous comparison between models and better insight into variations in their performance across different data splits, especially as computational resources become more available.

Expanding the unseen data with a more diverse participant pool can be used to assess further the model’s generalisability across a broader range of signers while applying quantitative metrics consistently across all datasets, including the non-signer set, will ensure that our comparisons remain fair and statistically sound.

By combining these efforts, the project could be enhanced to offer a universally applicable tool that bridges communication gaps for the deaf community worldwide while pushing the boundaries of innovation in sign language AI.

## Figures and Tables

**Table 1:** Arabic Sign Language Sentence Recognition (ArSLSR) dataset.

# of Repetitions	Sentence (English)	Sentence (Arabic)
104	can_you_help_me	هل تساعدني؟
102	wish_you_luck_in_work	أتمنى لك التوفيق في العمل
100	wish_you_good_journey	أتمنى لك رحلة موفقة
100	wish_you_good_vacation	أتمنى لكم إجازة سعيدة
103	please_quickly	لو سمحت بسرعة
100	Explain_to_me_again	اشرح لي مرة أخرى
104	sorry_I_can’t_stay	آسف لا أستطيع البقاء
100	sorry_for_being_late	آسف لتأخري
105	i_think_you_are_wrong	أظن (أعتقد) أنك مخطئ
100	repeat_what_you_said	أعد ما قلت
100	free_or_busy	الآن مشغول أم فاضي؟
100	happy_to_know_you	أنا سعيد بمعرفتك
100	i_disagree	أنا غير موافق
104	i_agree	أنا موافق
103	i_would_like_to_meet_you	أود مقابلتك
100	any_service?	أي خدمة؟
100	come_quickly	تعال بسرعة
102	good_bey	رافقتك السلامة
100	thanks_for_your_concern	شكراً على اهتمامك
101	happy_new_year	كل عام وأنت بخير
100	how_can_i_call_you	كيف يمكنني الاتصال بك؟
100	wait_please	لحظة من فضلك
100	lets_go_swim	لنذهب للسباحة
105	please_speak_slowly	لو سمحت تكلم ببطء
102	doesnt_matter	ليس مهم
104	whats_your_name	ما اسمك؟
100	what_about_going_for_a_walk	ما رأيك في أن تقوم بنزهة؟
100	this_is_nbelievable	هذا لا يصدق
100	can_i_take_from_your_time	هل لي أن أخذ من وقتك؟
110	i_have_to_go	يجب أن أذهب

**Table 2:** Test results for the TCN model using new videos (unseen data).

Input Sentence	Predicted Class with TCN	Confidence Score
اتمنى لك التوفيق في العمل (wish you luck in work)	اتمنى لك التوفيق في العمل (wish you luck in work)	0.85
آسف على التأخير (sorry for being late)	آسف على التأخير (sorry for being late)	1
شكرا على اهتمامك (thanks for your concern)	شكرا على اهتمامك (thanks for your concern)	1
لا يهم (it doesn’t matter)	لا يهم (it doesn’t matter)	0.86
هل يمكنك مساعدتي (could you help me)	هل يمكنك مساعدتي (could you help me)	0.98

**Table 3:** Result of evaluation of BiLSTM with attention model using unseen data.

Input Sentence	Predicted Class with BiLSTM and Attention	Confidence Score
اتمنى لك التوفيق في العمل (wish you luck in work)	اتمنى لك التوفيق في العمل (wish you luck in work)	0.65
آسف على التأخير (sorry for being late)	آسف على التأخير (sorry for being late)	1
شكرا على اهتمامك (thanks for your concern)	شكرا على اهتمامك (thanks for your concern)	1
لا يهم (it doesn’t matter)	لا يهم (it doesn’t matter)	0.83
هل يمكنك مساعدتي (could you help me)	هل يمكنك مساعدتي (could you help me)	0.86

**Table 4:** Sample evaluation table for unknown gesture detection.

Input Video Description	Actual Gesture	Predicted Class	Confidence Score	Final Output
A person waving both hands rapidly	Unknown	هل يمكنك مساعدتي (could you help me)	0.42	Unknown Gesture
Drawing a circle in the air	Unknown	شكرا على اهتمامك (thanks for your concern)	0.36	Unknown Gesture
Sign similar to “شكرا على اهتمامك (thanks for your concern)”	Unknown	شكرا على اهتمامك (thanks for your concern)	0.59	Unknown Gesture

**Table 5:** Key differences between models.

Criterion	TCN Model	Enhanced BiLSTM with Attention Model
Accuracy (ArSLSR Dataset)	0.995	Initially: 0.96 After enhancements: 0.99
Precision (ArSLSR Dataset)	0.99	Initially: 0.97 After enhancements: 0.99
Recall (ArSLSR Dataset)	1.00	Initially: 0.96 After enhancements: 0.99
F1-Score (ArSLSR Dataset)	0.99	Initially: 0.96 After enhancements: 0.99
Accuracy (External Dataset)	0.97	Initially: 0.26 After enhancements: 0.97
Precision (External Dataset)	0.99	After enhancements: 0.98
Recall (External Dataset)	0.99	After enhancements: 0.97
F1-Score (External Dataset)	0.99	After enhancements: 0.97
Generalisation	Excellent: Maintained high performance across both internal and external datasets.	It had poor generalisation initially; it significantly improved after enhancement.
Evaluation of Non-Signer Videos	High accuracy with confidence scores mostly near 1.	It was mostly accurate but with lower confidence scores, particularly visually similar signs.
Speed of Running	Running on ArSLSR took 17 min. Running on the external dataset took 41 min.	Initially: Running on ArSLSR took 52 min. Running on the external dataset took 58 min. After enhancements: Running on ArSLSR took two hours. Running on the external dataset took six hours.
Confusion Matrix Observations	There were a few misclassifications, mostly between sentences with visually similar gestures.	Initial confusion with external datasets; improved significantly with enhancements.
Adaptability	Robust performance, even with unseen non-signer data.	Improved adaptability after incorporating Conv1D and attention mechanisms.
Strengths	a. Strong temporal modelling with convolutional layers. b. Consistent performance across datasets. c. High confidence in predictions.	a. Enhanced contextual understanding with an attention mechanism. b. Improved feature extraction with Conv1D.
Weaknesses	There is a slight difficulty in distinguishing between very similar signs.	Initially, poor external dataset performance; enhancements improved, but still less confident than TCN.
Architectural Complexity	Simpler, with convolutional layers efficiently handling temporal dependencies.	It is more complex due to the addition of Conv1D, attention mechanisms, and BiLSTM layers.

## Data Availability

The data presented in this study are available on request from the corresponding author.
